# Exploring Indicators for Training Load Control in Young Swimmers: The Role of Inspiratory Spirometry Outcomes

**DOI:** 10.3390/jfmk9010053

**Published:** 2024-03-18

**Authors:** Adrián Feria-Madueño, Nuno Batalha, Germán Monterrubio-Fernández, Jose A. Parraca

**Affiliations:** 1 Department of Physical Education and Sport, University of Seville, 41013 Seville, Spain; 2 Departamento de Desporto e Saúde, Escola de Saúde e Desenvolvimento Humano, Universidade de Évora, 7004-516 Évora, Portugal; nmpba@uevora.pt (N.B.); jparraca@uevora.pt (J.A.P.); 3 Comprehensive Health Research Centre (CHRC), Universidade de Évora, 7004-516 Évora, Portugal; 4 Cardenal Spínola CEU University Studies Center, 41930 Bormujos, Spain; gmonterrubio@ceu.es; 5 Club Natación Mairena del Aljarafe, Mairena del Aljarafe, 41927 Seville, Spain

**Keywords:** peak inspiratory flow, countermovement jump, swimming, fatigue, performance

## Abstract

One of the most important implications of technology in swimming is to control training loads. Lactate control, video-analysis of the technique or the assessment of specific actions, i.e., the vertical jump, have helped to provide load adaptation indicators in swimmers in recent decades. However, these indicators have led to a longer application time, due to their indirect procedure and the need to analyze each variable. The aim of this study was to analyze whether inspiratory spirometry values can serve as a training load control tool in swimmers. Countermovement jump (CMJ), Inspiratory Force Index (S-INDEX) and Peak Inspiratory Flow (PIF) were evaluated with a load of 3 cm H_2_O before, during and after performing a swimming performance test (critical speed test: specific warming up, 400 m and 100 m freestyle). Positive correlations were found between S-INDEX and jump height after warm-up, after 400 m and at the end of 100 m (Spearman = 0.470, R^2^ = 0.280; Spearman = 0.508, R^2^ = 0.392; Spearman = 0.458, R^2^ = 0.359, *p* < 0.05, respectively). Moreover, positive correlations were also found between PIF and jump height at the same evaluated moments (Spearman = 0.461, R^2^ = 0.305; Spearman = 0.493, R^2^ = 0.386; Spearman = 0.454, R^2^ = 0.374, *p* < 0.05). Both the S-INDEX and the PIF could serve as useful tools for swimmer load control, allowing coaches to make more immediate decisions.

## 1. Introduction

The positive effect of physical conditioning on biomechanical aspects, neural adaptations of the upper and lower limbs and, particularly, the improvement in muscular strength, has recently been demonstrated as one of the most decisive contributions to the performance of swimmers [1]. Specifically, resistance training has been a major controversy in recent decades, influencing not only training planning and the type of load, but also the relationship it has with important elements like starts, turns or swimming speed [2]. Despite this controversy, the relevance of swimmers’ reaching certain levels of strength to their performance is evident, since strength training in swimmers seems to have a direct impact on performance [3,4]. However, endurance is an essential prerequisite for performance in swimming. The design of the training volume should be one of the most determining objectives in the planning of coaches and physical trainers. Authors such as Haycraft and Robertson [5] have analyzed concurrent swimming training, concluding that the accumulated volume should not exceed 5000 m of training per day, due to the negative impact of the effects of strength training on the system. In order to control training loads, swimming has traditionally been one of the sports where performance control, distance or the achievement of specific times have been tested to achieve specific training objectives. Currently, one of the most important implications of technology in swimming is the ability to control training loads. The lactate control, the video-analysis of the technique [6] or the assessment of specific actions such as a vertical jump have served as indicators of adaptation to the load in swimmers [2]. In recent years, the assessment of vertical jump has gained importance due to its correlation with sports performance in general and especially in actions that require strength and power of the lower body [7,8]. However, these indicators require a longer application time due to their indirect procedure and the need to analyze each variable. Authors such as Calderbank et al. [9] demonstrated the association between vertical jumps and dive distance in swimmers. Nuzzo et al. [10] found a correlation between dynamic multi-joint dynamic tests of strength, expressed through body mass, and countermovement jump (CMJ) performance. In line with explosive actions that fundamentally compromise the neuromuscular power generation system, numerous authors have concluded that the CMJ can be used to assess and predict performance in starts and turns in swimming and that it should be included in all dryland training routines [11,12]. Several authors have contributed valuable insights regarding the CMJ as a means of training load control. Notably, Sirieiro et al. [13] recognized the CMJ as a specific exercise for assessing the impact of training set configuration on young swimmers. Moreover, Strzala et al. [14] made a compelling discovery, suggesting the CMJ’s utility as a parameter for regulating training loads among elite competitive swimmers. Their work establishes a correlation between CMJ height and enhanced performance in the 100 m freestyle. Additionally, at 50 m distance, Zaras et al. [15] employed the maximum CMJ height to monitor the influence of swimming this distance on any alterations in power output during a vertical jump. These findings collectively underscore the multifaceted applications of the CMJ in evaluating and controlling training loads in the context of swimming performance.

Despite the fact that the CMJ seems to be a reliable indicator for performance control in swimmers, one of the main problems is the specificity of the test, as well as the feasibility of performing it both during swimming training and in competition. One of the methodologies that is currently attracting attention among training swimmers is inspiratory muscle training (IMT). Karsten et al. [16] suggest that a linear IMT represents an effective tool to improve inspiratory muscle strength and performance in athletes. Kilding, Brown and McConnell [17] concluded that IMT has a beneficial effect on performance in 100 m and 200 m swimming events, something also associated with a clear reduction in perceived exertion. These contributions are reinforced by recent research that reflects an increase in the Maximum Inspiratory Force Index (S-INDEX), ventilation and Maximum Inspiratory Flow (PIF), which positively influences the swimming performance of young swimmers [18]. Despite this, there is little evidence of the role of IMT as a training load control tool for swimmers. Therefore, the objective of this study was to analyze whether inspiratory spirometry values can serve as a load control tool for swimmers.

## 2. Materials and Methods

### 2.1. Subjects

The sample in the present study consisted of 30 nationally competitive swimmers (17 women; 13 men). All participants were familiarized with the CMJ test, as well as with inspiratory spirometry and with the performance test that was used (Critical Swimming Speed Test) in the four weeks prior to the intervention. Nevertheless, none of the swimmers had previous experience with the IMT methodology as a training method.

### 2.2. Procedure

The protocol was based on the implementation of a Critical Swimming Speed (CSS) or Maximum Aerobic Speed (MAS) test. The participants used a standardized warm-up that was divided into two parts: one dry and one in water. The dry warm-up consisted of 5 min of joint mobility and 30 CMJ with verbal instructions from the investigators. In the water, all subjects developed a standardized warming up.

**Figure 1:**
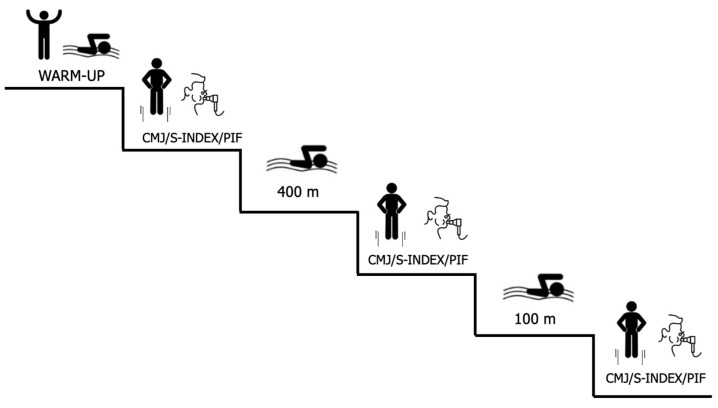
Assessment sequence for each athlete. CMJ = Countermovement jump; S-INDEX = Inspiratory Force Index; PIF = Peak Inspiratory Flow PIF.

After such practice, each subject performed a CMJ, immediately followed by an inspiratory spirometry assessment. Next, each athlete performed a CSS test. After the first 400 m freestyle corresponding to the test, the CMJ test and inspiratory spirometry were repeated. Finally, the swimmers performed the last 100 m freestyle corresponding to the test, and immediately after, a third evaluation repeating the CMJ and inspiratory spirometry (Figure 1).

All the participants signed their informed consent before beginning any evaluation test of the present study, according to the Declaration of Helsinki. The experimental procedure was approved by the Institutional Review Board of the Mairena del Aljaraje Swimming Club (approval code: 20225483; approval date: 15 February 2022).

### 2.3. Anthropometric Measures

The day before the evaluations, the swimmers’ body mass, height, leg length and hip height were evaluated in 90° flexion. A scale and a measuring tape were used to obtain the anthropometric data. The measurement followed the standards established by the International Society for the Advancement of Kinanthropometry (ISAK), and all measurements were recorded by an expert anthropometrist (Level 1 ISAK practitioner).

### 2.4. Vertical Jump

The CMJ was performed for the evaluation of the vertical jump. The swimmer was placed in an area close to the pool, three meters away from the researcher, who controlled a recording device (iPad digital device, iOS 15.3.1, Apple Inc., Cupertino, CA, USA). Subsequently, the video was processed using the Myjump 2 App [19]. The variables evaluated in the CMJ were jump height, flight time, speed, strength and power.

### 2.5. Inspiratory Spirometry

**Figure 2:**
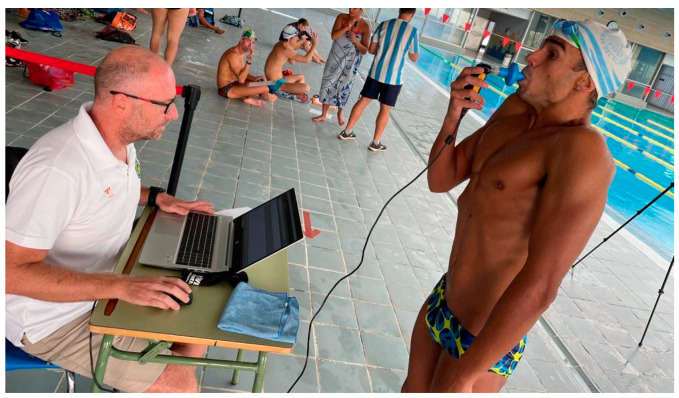
Athlete performing the inspiratory spirometry evaluation.

All swimmers performed a test related to the IMT methodology. The PowerBreathe K5 device (POWERbreathe K-5; Technologies Ltd., Birmingham, UK) was used to perform the inspirations and the data were processed with the Breathelink (Breathelonk Ltd., Cambridge, UK) immediate biofeedback software [20]. The protocol of inspirations carried out by each swimmer was five maximum inspirations, obtaining data from the S-INDEX and PIF (Figure 2) with a load of 3 cm H_2_O.

The S-INDEX is a measure of inspiratory muscle strength derived from the Peak Inspiratory Flow (PIF). This is a predictive value of the Maximum Inspiratory Power (MIP). The strength index calculation is based on the standard inspiratory muscle force–velocity relationship and its result is rated from poor to excellent [21]. S-INDEX and MIP have a strong correlation and good concordance, indicating that both are capable of evaluating the inspiratory muscle strength of healthy individuals [22]. The PIF consequently evaluates the ability of the inspiratory muscles to contract rapidly and overcome the resistance and elasticity inherent in the respiratory system. The inspiratory musculature in action is transduced into a force–velocity relationship; therefore, the inspiratory flow shows a reduction in all lung volumes in response to a weakness of the inspiratory musculature. Improvements in inspiratory muscle strength can be observed by monitoring changes in peak inspiratory flow (PowerBreathe^©^ BreatheLink K5 operating manual).

### 2.6. Critical Swimming Speed Test

The CSS is the maximum speed that can be maintained for a prolonged period of time without exhaustion [23], corresponding to the speed at the maximal lactate steady state (MLSS). This is expressed as the slope of the regression line relating to the swim distance and the corresponding swim time of a series of maximal effort time trials. However, in running, cycle ergometer and swimming, this relationship cannot be described by a single linear relationship [24]. The slope of the time–distance relationship depends on the range of exhaustion times, and therefore the number and specific duration of the exercises carried out. To calculate the CSS, the specific duration of the exercise must be defined and not chosen arbitrarily [25].

The swimmers, after being familiarised with the test, performed the distances according to the protocol. The 400 m and 100 m freestyle trials were set as test distances, with an intermediate recovery, which included the CMJ and IMT assessments that were conducted without the interference of the mentioned recovery.

### 2.7. Statistical Analysis

All data are shown as mean and ± SD. To analyze the parametricity of the data, a Shapiro–Wilk test was developed. To establish differences between variables, repeated measures ANOVA and Bonferroni post hoc were used. Spearman’s Rho was used to analyze the correlations between variables (*p* ≤ 0.05). To study the strength of these correlations, a linear regression was used. All statistical processing was performed with Jamovi software version 2.3.21 (Jamovi project, Sydney, Australia).

## 3. Results

The descriptive characteristics, together with the anthropometric measurements of the sample, are reflected in Table 1.

### 3.1. Countermovement Jump (CMJ)

The descriptive results, the differences between groups and the differences by sex during the three CMJs are shown in Table 2. 

As can be seen in the table, in the second CMJ achieved the highest results of all the variables with respect to the former and latter vertical jump. When the first is compared with the third, CMJ 3 that achieves the highest values for all the variables analyzed throughout the jump. Regarding height, the differences were significant between CMJ 2 compared to CMJ 3 (*p* < 0.001). Regarding CMJ height, males showed significantly higher values than females (*p* < 0.001) when comparing CMJ 2 with CMJ 3 (males: CMJ 2 = 34.7 ± 5.73; CMJ 3 = 31.8 ± 5.63; females: CMJ 2 = 27.3 ± 5.83; CMJ 3 = 25.4 ± 5.61).

### 3.2. Inspiratory Spirometry

The descriptive results of the three inspiratory spirometry executed by the swimmers are presented in Table 3.

As can be seen in the table, the second spirometry showed the highest results in both S-INDEX and PIF. Both variables presented significant differences between the first evaluation and the second (*p* ≤ 0.05). However, neither S-INDEX nor PIF presented significant differences between the second and third evaluation (*p* > 0.05). Regarding sex differences, in the S-INDEX, there were significant differences (*p* < 0.001) between the first and second evaluations (males: E1 = 135 ± 31.1; E2 = 148 ± 32.3; females: E1 = 85.0 ± 13.7; E2 = 87.0 ± 12.7). Similarly, there were significant differences (*p* = 0.021) between the first and third evaluations (males: E3 = 147 ± 31; females: E3 = 85.5 ± 16.1). Again, in relation to sex differences, in the PIF there were significant differences (*p* < 0.001) between the first and second evaluations (males: E1 = 7.36 ± 1.38; E2 = 7.98 ± 1.60; females: E1 = 4.86 ± 0.75; E2 = 4.96 ± 0.69). Along the same line, there were significant differences (*p* = 0.009) between the first and third evaluations (males: E3 = 7.95 ± 1.43; females: E3 = 4.88 ± 0.86).

**Table 2:** Countermovement jump results in the three trials.

	CMJ 1 (Mean ± SD)	CMJ 2 (Mean ± SD)	CMJ 3 (Mean ± SD)	Group Differences				Sex Diferences			
				*p* Value	Bonferroni			*p* Value	Bonferroni		
					CMJ 1–CMJ 2	CMJ 2–CMJ 3	CMJ 1–CMJ 3		CMJ 1–CMJ 2	CMJ 2–CMJ 3	CMJ 1–CMJ 3
Strength (N)	1216 ± 263	1254 ± 267	1247 ± 250	0.065	0.081	1.000	0.171	0.440	0.114	1.000	0.247
Power (W)	1480 ± 489	1569 ± 488	1543 ± 441	0.100	0.101	1.000	0.332	0.546	0.138	1.000	0.452
Height (cm)	29.5 ± 7.77	30.5 ± 6.78	28.2 ± 6.39	<0.001	0.209	<0.001	0.103	0.043	0.296	<0.001	0.035
Speed (m/s)	1.19 ± 0.16	1.23 ± 0.14	1.22 ± 0.13	0.046	0.098	1.000	0.137	0.317	0.141	1.000	0.202

SD: standard deviation; CMJ: countermovement jump; N: Newtons; W: Watts; cm: centimeters; m/s: meters per second.

**Table 3:** Inspiratory spirometry.

	E1 (Mean ± SD)	E2 (Mean ± SD)	E3 (Mean ± SD)	Group Differences				Sex Diferences			
				*p* Value	Bonferroni			*p* Value	Bonferroni		
					E1–E2	E2–E3	E1–E3		E1–E2	E2–E3	E1–E3
S-INDEX (cmH_2_O)	107 ± 33.8	112 ± 37.7	111 ± 36.2	0.004	0.006	1.000	0.093	0.008	<0.001	1.000	0.021
PIF (cmH_2_O)	5.94 ± 1.54	6.21 ± 1.89	6.15 ± 1.90	0.004	0.007	1.000	0.059	0.007	<0.001	1.000	0.009

SD: standard deviation; S-INDEX: strength index (maximum inspiratory power); PIF: peak inspiratory flow; cmH_2_O: centimeters of water pressure; E1: first spirometry; E2: second spirometry; E3: third spirometry.

### 3.3. Critical Swim Speed

The Critical Swim Speed (CSS) was used to control the performance of the swimmers. Both the speed and the times of the 400 m and the 100 m freestyle were evaluated, as well as the critical speed (Table 4).

### 3.4. CMJ Ratio and Inspiratory Spirometry

**Figure 3:**
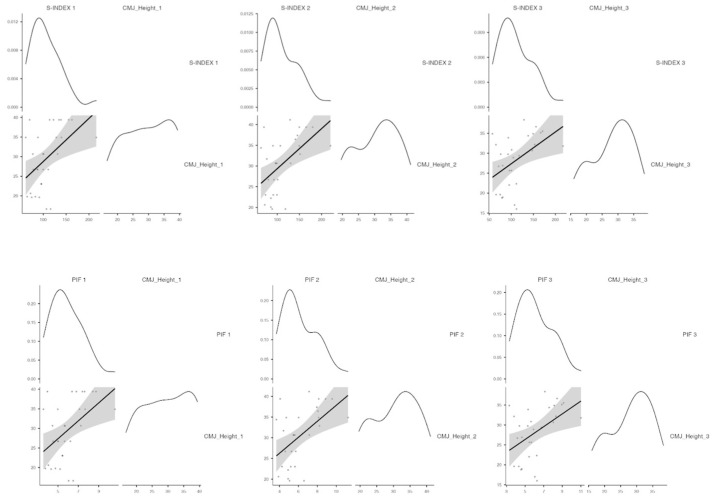
Correlations between the S-INDEX and PIF and the height in the CMJ test at the three evaluated points. CMJ = Countermovement jump; S-INDEX = Inspiratory Force Index; PIF = Peak Inspiratory Flow PIF.

The relationship between the CMJ and inspiratory spirometry variables was evaluated (Figure 3).

Positive correlations were found between the S-INDEX and the jump height after the warm-up, after the 400 m and at the end of the 100 m (Spearman = 0.470, R^2^ = 0.280; Spearman = 0.508, R^2^ = 0.392; Spearman = 0.458, R^2^ = 0.359, *p* < 0.05, respectively). Positive correlations were also found between the PIF and the jump height at the evaluated points (Spearman = 0.461, R^2^ = 0.305; Spearman = 0.493, R^2^ = 0.386; Spearman = 0.454, R^2^ = 0.374, *p* < 0.05).

### 3.5. Inspiratory Spirometry and Performance Relationship

**Figure 4:**
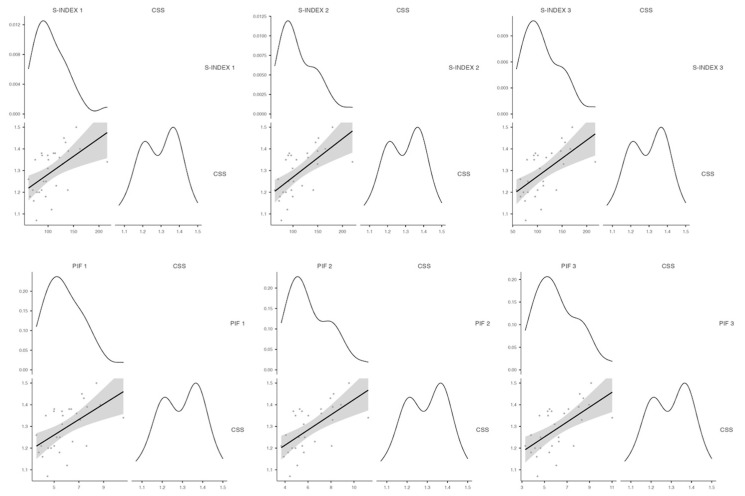
Correlations between the S-INDEX and PIF and the performance in the CSS test at the three evaluated points.

The relationship between the inspiratory spirometry and performance in the CSS test is shown in Figure 4.

A positive correlation was obtained between the ventilatory parameters of S-INDEX and PIF and the CSS after the warm-up (Spearman_S-INDEX = 0.592, Spearman_PIF = 0.591, R^2^ = 0.233; *p* < 0.05), after the 400 m (Spearman_S-INDEX = 0.658, Spearman_PIF = 0.636, R^2^ = 0.280; *p* < 0.05) and after the 100 m (Spearman_S-INDEX = 0.616, Spearman_PIF = 0.610, R^2^ = 0.221; *p* < 0.05).

### 3.6. CMJ and Performance Relationship

The correlation between CMJ height and CSS was analyzed. We found that there is a positive correlation between both variables in the first jump (Spearman = 0.538, R^2^ = 0.281; *p* = 0.002), in the second (Spearman = 0.594, R^2^ = 0.318; *p* < 0.001) and in the third jump (Spearman = 0.600, R^2^ = 0.333; *p* < 0.001). 

## 4. Discussion

The aim of this study was to analyze inspiratory spirometry values and their relationship with the control of training loads in swimmers. To the best of our knowledge, this is the first study that shows a correlation between inspiratory spirometry parameters related to CMJ to determine whether they could serve as load control in young swimmers. Another of the most important findings of the present study was the correlation between the inspiratory spirometry parameters and performance, as evaluated using a maximum aerobic speed test. Both the S-INDEX and the PIF can serve as useful indicators of the level of sports performance in swimmers.

The findings in the present study are consistent with the findings of Karsten et al. [16], who conducted a systematic review and meta-analysis focusing on the effects of muscle breathing training with linear load devices on athletic performance and cardiopulmonary function in athletes. The results of the review suggest that respiratory muscle training with technological devices may have positive effects on athletic performance and cardiopulmonary function in athletes. Authors such as Pérez-Olea et al. [26] confirmed that swimmers who showed higher levels of strength had a higher performance, specifically in anaerobic critical velocity [27]. 

In contrast to earlier findings, however, no evidence of a relationship between performance exercise and CMJ was detected. The CMJ has become a good indicator of sports performance in swimmers. As reflected by Calderbank et al. [9], there is a close association between CMJ ability and the maximum force manifested during the diving distance in swimmers. Although our study did not assess this aspect of the swimmers’ performance, a significant relationship between jump height and critical speed was found. Our results agree with those presented by Zaras et al. [15], who recently detailed a significant correlation between the power generated in the CMJ and 50 m performance in experienced young swimmers. In addition, our results agree, in part, with what was described by Yañez-Sepulveda et al. [18], who studied the action of IMT on the performance of short-distance swimmers, specifically, the effect in 50 m, 100 m and 200 m swims. These authors also based the analysis of the IMT on the S-INDEX and the PIF. The authors concluded that there was a correlation between the S-INDEX and the PIF concerning performance in the 50 m and 100 m tests, but it was not significant in the 200 m test. Despite not finding a correlation in the 200 m test, the study showed a performance improvement when the experimental group that did train with IMT was compared to a control group. Other authors found an improvement in performance in the 200 m after having trained using the IMT methodology [17]. In brief, a significant relationship between respiratory training and performance has recently been reported in swimmers [28]. According to our results, there is a correlation between swimmers with higher levels of S-INDEX and PIF and performance as evaluated by means of the CSS. There are several possible explanations for these results. For example, the IMT could produce pulmonary adaptations in swimmers [29]. However, the purpose of the present study was not to assess the effect of IMT training on performance but to consider its use as a load control tool. 

A considerable amount of literature has been published on the role of the CMJ as a good predictor in the control of training load in swimmers. In line with explosive actions or actions that are fundamentally addressed to the neuromuscular power generation system, authors such as Carvalho et al. [11] concluded that the CMJ can be used to assess and predict performance in swimming starts and turns and should be included in all dryland fitness routines. Our results reflect a significant correlation between the CMJ values (strength, power, height and flight speed) with the S-INDEX and the PIF. As far as we know, this is the first study that analyzes this correlation, presenting significant findings on the possibility of controlling training loads in swimmers. Our results could have an important practical application for coaches because both S-INDEX and PIF could be used as training control tools. A possible explanation for the results may be based on the ergogenic factors of swimming, since, as stated by Kilding, Brown and McConnell [17], immersion in water forces swimmers to expand their chest wall against greater pressure, while also increasing inspiratory muscle contraction velocity and tidal volume (VT), which could lead to muscle fatigue.

Finally, some limitations that are present in the current study are acknowledged. The primary constraint pertains to the sample size, comprising only 30 subjects. Despite their homogeneity in terms of age and years of practice, gender introduces a potential sensitivity variable that may influence the interpretation of results. Nevertheless, gender differences among the variables of CMJ and inspiratory spirometry have been investigated. Additionally, the identified correlations, while positive, appear to exhibit relatively low coefficients of determination (R^2^). Therefore, it is recommended that the data are interpreted cautiously. Future studies should address these limitations and aim to rectify them.

## 5. Conclusions

Our results suggest that the evaluation of both the S-INDEX and the PIF could be a useful indicator of the level of sports performance and load control in swimmers, providing coaches with useful and feasible tools to use during training and allowing for more immediate decision-making for the trainers. 

## Figures and Tables

**Table 1:** Characteristics of the participants (*n* = 30).

	Mean ± SD
Age (years)	17.0 ± 2.20
Height (cm)	171 ± 10.6
Leg Length (cm)	109 ± 5.99
Heigh_90° (cm)	75.8 ± 4.49
Body Mass (Kg)	63.9 ± 9.68
Practice (years)	8.93 ± 2.83

cm: centimetres; Kg: kilograms; 90°: hip height at 90° of flexion; SD: standard deviation.

**Table 4:** Swimmer performance data.

	Time 400 m (s) (Mean ± SD)	Speed 400 m (m/s) (Mean ± SD)	Time 100 m (s) (Mean ± SD)	Speed 100 m (m/s) (Mean ± SD)	CSS (m/s) (Mean ± SD)
Data	299 ± 25.8	1.35 ± 0.11	65.44 ± 6.83	1.51 ± 0.18	1.29 ± 0.10

SD: standard deviation; CSS: critical swim speed; s: seconds; m/s: meters per seconds.

## Data Availability

Dataset available on request from the authors.
